# Arterial abnormalities in the hands of workers with vibration white fingers – a magnetic resonance angiography case series

**DOI:** 10.1186/s12995-021-00319-x

**Published:** 2021-07-29

**Authors:** Per Vihlborg, Karim Makdoumi, Hana Gavlovská, Sverre Wikström, Pål Graff

**Affiliations:** 1 Department of Occupational and Environmental Medicine, Faculty of Medicine and Health, Örebro University, SE 701 82 Örebro, Sweden; 2 Odensbackens Health Center, Örebro, Sweden; 3 Departement of geriatrics, Faculty of Medicine and Health, Örebro University, SE 701 82 Örebro, Sweden; 4 School of Medical Sciences, Örebro University, Örebro, Sweden; 5 Department of Ophthalmology, Faculty of Medicine and Health, Örebro University, Örebro, Sweden; 6 Department of radiology, Örebro University Hospital, PO Box 1613, SE-701 16 Region Örebro County, Sweden; 7 National Institute of Occupational Health (STAMI), Oslo, Norway

**Keywords:** Hand-arm vibration, Vibration white finger, Raynaud’s syndrome, Vascular abnormalities

## Abstract

Vibration white finger (VWF) is a complication from exposure to hand-arm vibrations. Poor knowledge of the pathophysiology of VWF means that making an accurate prognosis is difficult. Thus, a better understanding of VWF’s pathophysiology is of importance.

The purpose of this study was to investigate whether there were arterial abnormalities in the hands in patients with VWF and a positive Allen’s test, using ultrasound and MRA imaging.

This was a case series where arterial abnormalities in the hands were investigated in ten participants with VWF and using prolonged Allen’s test (> 5 s). The participants had an average vibration exposure of 22 years and underwent Doppler ultrasound and Magnetic Resonance Angiography (MRA) to check for arterial abnormalities.

The participants had VWF classified as 1–3 on the Stockholm workshop scale. Ultrasound and MRA identified vascular abnormalities in all participants, the predominant finding was missing or incomplete superficial arch. Also, stenosis was identified in four participants.

This study reveals a high proportion of arterial stenosis and abnormalities in patients with VWF and a prolonged Allen’s test.

**Supplementary Information:**

The online version contains supplementary material available at 10.1186/s12995-021-00319-x.

## Introduction

Vibration white finger (VWF) is a common complication that can arise from working with hand-held vibrating tools and it is classified as a secondary form of Raynaud syndrome (RS) [4, 24, 28]. A characteristic of RS is reduced blood flow, caused by the spasm of arteries, which leads to blanching of the fingers but rarely the thumb when exposed to the cold [16, 22]. The symptoms of RS caused by vibrations, i.e., VWF, typically appear after several years of exposure to hand-arm vibrations. Both the intensity and the duration of vibration exposure are associated with increased risk of VWF [4, 13]. The severity of VWF is usually classified according to the Stockholm workshop scale (SWS), based on the extent of the blanching and the frequency of symptoms [14].

The pathophysiology for VWF is not fully understood. In addition to increased arterial constriction [12] and a reduced ability of digital vasodilation [9, 26]. It is also suggested that exposure to vibrations can lead to direct damage to the endothelial walls of the vessels and an inflammation/immune response, resulting in vascular obstructions [17, 21, 26].

An Allen’s test is routinely performed to identify reduced blood flow to the hand in either the ulnar or the radial artery by testing vascular reactivity through fist clenching [1]. In studies on workers exposed to vibration with Raynaud syndrome (or VWF), a prolonged Allen’s test was found in about 50% of individuals with symptoms of VWF [2]. Also, platers exposed to vibration with VWF had an approximately four times higher risk of a prolonged Allen’s test [23].

The authors concluded that an Allen’s test is of value in the clinical examination of suspected VWF. Patients with VWF and pathological Allen’s tests are commonly referred for further investigation through Doppler ultrasonography. The obstruction can be visualised and further action can be taken such as angiography and surgery [8, 27].

Lately, Magnetic Resonance Angiography (MRA) methods have emerged, providing information comparable to conventional angiography [6].

In a case study, Poole et al. conducted MRA on the hands of three patients with VWF and arterial occlusions were found in all three of these [25]. This study suggested that workers with only one artery supplying the hand or with only one palmar arch may be at increased risk of progression. Thus, MRA of the hands in individuals displaying symptoms of VWF could give a valuable insight into which individuals should not be exposed to hand-transmitted vibration irrespective of their classification on the Stockholm workshop scale.

Today, the pathophysiology of VWF is otherwise mostly unknown and there is no specific treatment. There is also a lack of objective methods such as laboratory or imaging methods to help to diagnose VWF. The inadequate knowledge of the pathophysiology of VWF hinders the development of treatments and makes it difficult to provide an accurate prognosis for the patients. Therefore, it is important to gain a better understanding of VWF’s pathophysiology and identifying potential arterial abnormalities that might be targeted for intervention.

## Aim

The purpose of this study was to investigate if arterial abnormalities in the hands could be identified in patients with VWF and a positive Allen’s test using ultrasound and MRA imaging.

## Methods

The study was a case series of patients who were referred to the Department of Environmental and Occupational Medicine at Örebro University Hospital (USÖ) to assess hand-arm vibration symptoms (HAVS). The patients were referred to the USÖ from local occupational health services. The patients were asked to participate in the study if their symptoms met the eligibility criteria listed below. Oral and written informed consent was given by participants before data collection was initiated.

Inclusion criteria were: a) A history of vibration exposure and symptoms of vasoconstriction of the fingers when exposed to the cold (i.e., Raynaud syndrome) and b) A prolonged Allen’s test exceeding 5 s i.e., a positive Allen’s test [20].

Exclusion criteria were: being aged below 18 years, pregnancy, having an impaired liver, kidney or lung function, a history of cardiovascular disease other than isolated hypertension or hypercholesterolemia. Furthermore, patients with any previous allergic reaction to X-ray contrast or diabetes and taking any oral medication or insulin treatment were excluded from participation.

All study participants underwent a standardized clinical HAVS investigation, consisting of knowing their medical history, vibration exposure history, giving them a medical examination, blood analysis and Doppler ultrasonography.

The medical examination included a gross examination of the neck and shoulders, including the Roos (Elevated arms) Test to screen for thoracic outlet syndrome. Specific testing conducted of the hands involved Tinel’s and Phalen’s tests, followed by Allen’s test. The Allen’s test was performed by asking the patients to clench their fists 5 times to induce a constriction of the arteries in the palm. The test was considered normal if the blood flow returned to normal after opening the hand within 5 sec [1, 20].

Quantitative sensory testing was performed to verify the clinical diagnosis, consisting of a Vibration perception threshold, Touch perception, Grip strength and Thermal perception threshold.

Blood analyses included the hemoglobin concentration, leucocyte count, thrombocyte count, C-reactive protein, serum electrolytes, creatinine, aspartate aminotransferase and alanine aminotransferase, blood glucose levels, thyroid-stimulating hormone, cryoglobulins, rheumatoid factor and antinuclear antibodies.

The history of vibration exposure for all the participants was estimated by an occupational hygienist using calculations according to work history combined with a workplace measurement when possible. The estimated dose was divided into three categorise: Low (< 2.5 (m/s^2^) *year); Medium (2,5–4 (m/s^2^) *year) and High (> 4 (m/s^2^) *year).

Doppler ultrasound was conducted to visualise impairment in the blood supply from the specific artery. An experienced clinical physician conducted the examinations. The ultrasonography equipment used was GE logiq E9 (GE, Tampa FL USA) with probes GE ML 6–15 and GE L8-18i. The ulnar and radial arteries were examined distally in the forearm to identify any aneurysm, narrowing, or occlusion (assessment of flow rates and the vessel wall appearance). The dorsal part of the radial artery was followed down over the hand and the plantar branch was followed as far as possible to control the flow. The ulnar artery was followed along the hypothenar region and to the arcade. Flow measurements were taken at a uniform location in the arcade during compression of the radial artery and the ulnar artery, respectively. A normal flow measurement was defined as an increase in the arcade’s flow rate upon compression of the radial or ulnar artery on the contralateral artery. If the flow in the arcade was reduced upon compression, it was considered a sign of no arch connection, alternatively, an occlusion distal to the measuring point.

For MRA investigation, a GE 750w Discovery 3 T, GE Health care US with software version DW25 were used. The investigation was conducted with the patient lying on the back with their hand fixed in a wrist coil. Dotarem (Gothia medical Billdal Sweden) 0.2 ml/kg was used as a contrast.

The examination was conducted in four phases. Initially, a 3-plane localiser phase was conducted for the correct position of the hand in the wrist coil. Then, a sagittal PD (proton density) 5 mm anatomical picture series was carried out for planning the contrast phase. For the contrast phase, a coronal SUB Tricks 48 dynamic phase (a tissue subtracting series with 48 dynamic phases) was conducted for the contrast phase and artery to vein, which gives 48 stacks with different contrast phases. A rotation-MIP (Maximum intensity projection) was then carried out on the best phases to create a three-dimension angiogram. An experienced radiologist evaluated all the MRA examinations.

Finally, a Coronal 3D INHANCE VENC (velocity encoding) 40 phase was conducted to visualise arteries without contrast. This phase was not used to interpret the result, but to examine the possibility of not using any contrast in examinations.

The project was also approved by the Regional Ethical Review Board in Uppsala, Sweden (Dnr 2017/025).

## Results

The ten participants included in the study (Table 1) were all men and their age ranged from 21 to 65 years. Vibration exposure was on average for 22 years. The mean exposure per year varied from low (< 2.5 m/s^2^) in three individuals, between 2.5–4 m/s^2^ in five and above 5 m/s^2^ in two. None of the study subjects were active smokers, but four used snuff (non-smoking tobacco) and three were former users of tobacco products.

**Table 1:** Background data of the ten study participants

	N	%	Mean	Median	Range
Sex					
Male	10	100			
Female	0	0			
Age groups					
All	10	100	48	49	21–65
< 35	2	30			
36–50	3	30			
> 51	4	40			
Tobacco habits					
Non-tobacco user	3	30			
Snuff user	4	40			
Smoker	0	0			
Former tobacco user	3	30			
Years in vibration exposure					
All	10	100	22	20	2–43
< 10	1	10			
10–19	3	30			
20–29	3	30			
> 30	3	30			
Average yearly exposure					
Low < 2.5	3	30			
Medium 2.5–4	4	40			
High > 4	2	20			
SWS Vascular					
All	10				
0	0				
1	3				
2	5				
3	2				
SWS neurological					
All	10				
0	3				
1	6				
2	1				

Years in vibration exposure: in years, Average yearly exposure: Low (< 2.5 (m/s^2^) *year); Medium (2,5–4 (m/s^2^) *year) and High(> 4 (m/s^2^)*year). *SWS* Stockholm workshop scale

All participants had vascular symptoms according to the SWS for vascular and neurological symptoms. VWF or vascular symptoms ranged from stage 1 (distal phalanx blanching) to stage 3 (blanching down to the proximal phalanx) according to the Stockholm workshop scale, nine had bilateral VWF symptoms. Neurological symptoms were less frequent than vascular symptoms among the participants. Of the ten participants, three had no neurological symptoms, six had mild symptoms (classified as stage one according to the SWS) and only one had symptoms classified as SWS stage two. None of the study participants had a positive blood test for rheumatic disease that could explain the VWF.

Five persons reported localised Raynaud syndrome, i.e., symptoms localised to parts of the hand (Table 2). The Allen’s test was positive in all participants, five in the area corresponding to the ulnar artery and five in the radial artery. There was just slight blanching in one participant when compressing the wrist and the Allen’s test was difficult to perform (participant No. 5).

**Table 2:** Table of the individual cases on the exposure, symptom and findings, imaging shown in supplement file

No	Age	Year/exp	Average exposure	SWS neurological	SWS vascular	Localized Raynaud	Allen’s test	Ultrasound stenosis	Ultrasound abnormalities	MRA stenosis	MRA abnormalities
1	65	20	High	1	1	No	Ulnar	Radial artery stenosis	–	Stenosis in ulnar part of arch	Only deep arch, Ulnar corkscrew appearance
2	63	40	Medium	1	2	No	Radial		Reduced flow in the radial artery	Inadequate quality^a^	Inadequate quality^a^ Ulnar corkscrew appearance
3	60	43	Low	0	1	Digit 4–5	Ulnar	Ulnar artery occlusion		Ulnar artery occlusion radial artery stenosis	Only deep arch normal, with an abnormal variant of superficial arch that originates from the deep arch
4	59	30	Low	2	2	Digit 2–4	Ulnar	Ulnar artery stenosis, thin ulnar artery	Suspect flow by collaterals to arch	Ulnar artery stenosis	Only deep arch. Ulnar corkscrew appearance
5	49	22	Low	1	2	Digit 3–5	Ulnar	–	Only ulnar artery flow	–	Deep arch and abnormal superficial arch (duplicated)
6	42	14	Medium	0	3	Digit 2–3	Ulnar	Radial artery stenosis		–	Only deep arch and incomplete arch formation, Ulnar corkscrew appearance
7	35	12	Medium	1	2–3	No	Ulnar	Radial artery stenosis		Stenosis in ulnar part of arcus	Only superficial arch and stenosis or incomplete deep arch
8	32	14	Medium	1	2	No	Radial		Spastic vessel Atypical anatomy	–	Only one arch with atypical anatomy-Persisten Median Artery
9	21	2	Medium	0	1	No	Radial		Only ulnar artery flow	Thin radial artery and suspected vasospastic segment rather than stenosis	Only deep arch
10	49	20	Medium	1	2	No	Radial		Only ulnar artery flow	Stenosis or spastic segment in ulnar part of the arch	Deep arch and incomplete superficial arch

No: Participants, Year/exp.: Number of years of vibration exposure. Average exposure: Average yearly exposure Low (< 2.5 (m/s^2^)*year); Medium (2,5–4 (m/s^2^)*year) and High(> 4 (m/s^2^)*year). *SWS* Stockholm workshop scale. Localized Raynaud’s: If symptoms from Raynaud syndrome was localized at any part of the hands. Dig = which of the fingers 1–5 is affected. Allens: Radial = prolonged in radial supplied area. Ulnar = prolonged in ulnar supplied area. Ultrasound stenosis: suspected or visual stenosis on Doppler-Ultrasonography. Ultrasound abnormalities: anatomical abnormality or reduced blood flow without any suspected stenosis on Doppler-Ultrasonography. MRA Stenosis: suspected or visual stenosis on MRA. MRA abnormalities: anatomical abnormality or reduced blood flow without any suspected stenosis on MRA

^a^ Inadequate due to large hands

The ultrasound examinations identified an occlusion in the ulnar artery in one participant (hypothenar hammer) (participant no. 3) and five participants had suspected distal stenosis/occlusion at the arch level (participants No.1, 3, 4, 6 and 7). In addition to this, a suspected collateral flow to the palmar arch was found in one participant (participant No. 4). In five participants, ultrasound examinations revealed that no occlusion or stenosis was detected. However, in these cases, atypical anatomy was still suspected due to anomalous blood flow during a Doppler examination with arterial blood supply originating primarily from only one vessel to the arch (participants No. 2, 5, 8) and absent arterial radial flow was visualised in two participants (participants No. 9, 10).

Because of the relatively large hand-size, the MRA examination quality for one participant did not allow for assessments (participant No 2). MRA revealed stenosis in four participants and was consistent with the ultrasonography findings (participants No. 1, 3, 4, 7). The MRA indicated a narrowed segment in two participants where stenosis could not be ruled out although a vasospastic segment was concluded a more plausible explanation (participants No. 9, 10).

In six participants (participants No. 1, 3, 4, 6, 9 and 10), the MRA identified abnormal arterial anatomy, with a deep arch only and the absence of the superficial arch with a combined stenosis three (participants No. 1, 3 and 4). In one of these participants (No. 3) a superficial arch seemed to be present although it was connected from the middle of the deep arch. Also, in one participant, only the superficial arch was present (participant No. 7). Furthermore, the MRA showed abnormal anatomy in two examinations, with the presence of three arteries supplying the hand (participant No. 8) and a variant of duplicated superficial arches (participant No. 5), respectively. Four participants displayed an ulnar artery with a tortuosity or corkscrew appearance (participants No. 1, 2, 4 and 6).

When comparing ultrasound and MRA findings, it should be noted that ultrasonography compared to MRA could not differentiate whether the palmar arch was supplied by one or two arch (es) but enabled the detection of abnormalities in blood flow that were interpreted as a solitary ulnar arterial blood supply in three participants (participants No. 5, 9 and 10). In one participant, a suspected stenosis according to ultrasound was not observed on the MRI (participant No. 6).

## Discussion

In all ten patients with VWF and a prolonged Allen’s test who were included as participants in this study, structural arterial abnormalities of the hand were found according to MRA and/or ultrasound. This suggests the importance of arterial supply abnormalities in the pathophysiology of VWF.

Because of the study design, all participants had a prolonged Allen’s test and VWF (i.e., vascular) symptoms were more common than neurological symptoms. Usually, neurological symptoms are generally more common than vascular symptoms among workers exposed to vibration [24]. Earlier studies indicate that 15–37% of the general population have abnormalities in the superficial arterial arch of the hands [18, 19] compared to our study where all participants had imaging signs of any arterial abnormalities. There are also more recent indications highlighting the importance of vascular structural abnormalities in VWF. In an MRA case report by Poole et al., the three workers with VWF had vessel-occlusions, suspected to be caused by exposure to vibrations and the presence of vascular abnormalities in the form of nailfold capillaries has been demonstrated in RS [17, 25].

Furthermore, vibration-exposed workers with VWF have an increased frequency of a prolonged Allen’s test (about 50%) when compared to workers exposed to vibration without VWF [2, 23]. Impaired blood flow to the hands makes the arteries of the fingers more susceptible to cold exposure due to less warming because of less of an excess of warm blood and therefore, being more susceptible to cold-induced vasospasm. It might also be that the normal physiological constriction in cold weather produces a more pronounced decrease in circulation because of vascular abnormalities, which reduces the capability to maintain blood flow. Altogether, our findings are in line with previous research [17, 25]. It seems plausible that anatomical arterial abnormalities may, at least in part, be the cause of both VWF symptoms and the reduction of blood flow represented by the prolonged Allen’s test observed in this specific patient group and clinical setting. Hence, our data indicate that an underlying anatomical explanation for VWF should be suspected, at least when an Allen’s test is prolonged.

Other conditions that may present a clinical picture similar to Raynaud syndrome or VWF due to impaired blood flow are Thoracic outlet syndrome, Hypothenar hammer syndrome (HHS), Thenar hammer syndrome (THS) and Buerger’s disease, which are all associated with a partial or total blood flow impairment to the digits [7, 8]. HHS and THS are both caused by vascular obstruction, the ulnar artery (HHS), or the radial artery (THH), which is often secondary to trauma. These conditions can be accompanied by a whitening of the digits when exposed to cold and are difficult to distinguish from VWF, but may also contribute to its development [5, 7, 30]. In our participants, one (participant No 3) had signs of HHS on an MRA with an ulnar occlusion. Also, four (participants No 1, 2, 4 and 6) had tortuosity or a corkscrew appearance of the ulnar artery, which is a common radiological sign of HHS [3].

Interestingly, in this study, abnormalities found were mostly related to the superficial arterial arch. The thumb, which is usually most resistant to VWF and is rarely blanched except in severe cases gets its blood supply from the deep arch [6, 24]. This situation might be a protective factor for VWF of the thumb.

The correlation between ultrasound and MRA findings was low. Although ultrasound with a Doppler examination could detect the presence of anatomical abnormalities effectively, the indications regarding the specific location of stenosis were, in several cases, seemingly incorrect. Hence, it seems plausible that MRA could be a superior method to detect both the site of stenosis and underlying anatomical abnormalities in patients with VWF. In 90% of the patients examined in this study, an anatomical explanation could be detected using the magnetic resonance examination. This examination accurately locates the pathology and provides a wide range of information about vascular abnormalities, such as atypical vascular anatomy. We propose that this may improve diagnostic accuracy at earlier stages of the condition or in the future, even help to identify vibration-exposed subjects at risk of developing VWF. Since ultrasonography can also assess blood flow in vessels, it is unlikely that MRA should completely substitute an ultrasound examination in cases of VWF.

Conventional angiography is the gold standard for vascular abnormalities, but MRA yields comparable information about vascular anatomy, stenosis and obstruction [6]. Moreover, it is non-invasive and another potential advantage over conventional angiography is that MRA may identify connective tissue disorders, including vasculitis [6, 15]. On the other hand, conventional angiography is invasive, and complications from catheterisation make it less suited as a routine examination for VWF. However, MRA still uses intravenous gadolinium contrast [15, 25], but methods without contrast are under development [10, 11, 29]. In our study, the imaging from the non-contrast phase was inadequate for the study of arterial abnormalities.

A strength of this study is the thorough clinical examination of all study subjects included. Furthermore, the grading of symptoms and the assessment of the duration and intensity of exposure were carried out on an individual level. The patients originated from different regions of the country and worked in different companies, which is why it is unlikely that hereditary factors or particular local exposure influenced the outcome of the study. Also, the age in the group varied from 21 to 65 years, resulting in a mixed group representing vibration-exposed workers in a clinical setting.

Hence, we consider it an advantage by comparing the clinical symptoms with imaging of arterial blood flow displayed by two different techniques (ultrasonography and MRA). This enables a further understanding of the pathophysiology of VWF. Although vascular abnormalities were found in all patients, the design of the study cannot ascertain whether these were innate or acquired (because of vibration exposure). A prospective study has to be conducted with an MRA before the start of exposure to answer that question.

One limitation to this study is that although vascular abnormalities were found in all patients, the design of the study cannot ascertain whether these were innate or acquired (because of vibration-exposure). In order to verify causality, a prospective study would be required with angiographic examination before the start of exposure, but given the extended duration before the onset of symptoms, such a trial would be impractical. It would have been desirable with a randomised controlled study design. However, a randomised protocol would require a considerably larger patient cohort and would likely call for a multi-centre study to guarantee an adequate sample size. A control group, however, would have been especially beneficial for the interpretation of MRA examination results. Since the procedure is not customarily conducted in non-symptomatic subjects, there is limited knowledge about the average population’s anatomical variations without previous vibration exposure. We primarily selected the non-randomised protocol of this pilot study due to possible adverse effects of the phase contrast, which would be hard to motivate in a group of healthy control subjects. This study aimed at investigating whether there was an underlying anatomical pathology that could explain symptoms in VWF with a prolonged Allen’s test. There is some degree of potential selection bias due to the non-randomised inclusion, albeit that the consecutive inclusion of all patients fulfilled the study criteria during the period. On the other hand, according to their symptoms on the Stockholm workshop scale, few had severe symptoms.

There was no information on the use of gloves among the study participants. The use of gloves might reduce the risk for HHS, which is believed to be caused by repetitive blunt trauma that damages the vessels [1]. For arterial arch stenosis, there could be a protective factor in wearing gloves to decrease the grip pressure over the arterial arch in the hands, especially if the hand circulation depends on one arch.

Based on the findings of this study, we suggest that MRA is a suitable method to examine arterial abnormalities in the hands of patients with VWF and is complementary to ultrasound investigation. The method is relatively expensive, time-consuming, and associated with certain risks (due to intravenous contrast), although it is still considered safer than conventional angiography. In the future, the newly emerging techniques for non-contrast MRA [10] may change the cost-benefit balance of MRI in VWF. A more thorough and early investigation of patients’ hand arteries when there are signs and symptoms of VWF might be advantageous since our findings indicate a subgroup of VWF where symptoms depend on gross arterial abnormalities. This subgroup of VWF may thus, as already proposed [25], be advised to avoid exposure to hand-transmitted vibrations irrespective of their classification on the Stockholm workshop scale. Finally, some cases may benefit from the intervention of proximal stenosis.

In conclusion, this study reveals a high proportion of arterial stenosis and abnormalities in patients with VWF and a prolonged Allen’s test. We suggest that Magnetic Resonance Angiography could be beneficial, especially in combination with ultrasonography to better understand the underlying causes and arterial anatomy in patients with VWF. However, longitudinal studies comparing larger group of workers with different vibration exposures are needed to establish this method as a clinical tool.

## Supplementary Information

**Additional file 1: Figure S1.** Patient with prolonged Allen’s test on the ulnar side and 20 years of high vibration exposure. The ultrasound (US) showed suspected distal radial occlusion, and MRA shows only deep arch and stenosis in the junction with the ulnar artery. Ulnar artery shows a corkscrew appearance. **Figure S2.** Patient with 40 years of vibration exposure and prolonged Allen’s test on the radial. The US showed reduced flow from the radial artery MRA image quality was inadequate because of the size of the hands, making the use a wrist coil impossible instead examined in head coil. Ulnar artery shows a corkscrew appearance. **Figure S3.** Patient with prolonged Allen’s test on the ulnar side and 43 years of vibration exposure. US and MRA show an occlusion in the ulnar artery, and the MRA also shows a stenosis in the radial artery. Deep arch is present and a variant of superficial arch that originates from the deep arch. **Figure S4.** Patient with 30 years of vibration exposure and prolonged Allen’s test on the ulnar side. The US showed a thin ulnar artery. The MRA picture shows deep arch and stenosis in the junction of the deep arch to ulnar artery. Ulnar artery shows a corkscrew appearance. **Figure S5.** Patient with 22 years of low vibration exposure with Allen’s test prolonged ulnar. In this patient, the Allen’s test was difficult to perform. The compression of the wrist did not completely produce a paused blood supply. The US showed only ulnar flow, and MRA shows a variant with duplicated superficial arch and a normal deep arch. **Figure S6.** Patient with 14 years of vibration exposure and prolonged Allen’s test ulnar. The US indicated suspected a radial stenosis and the MRA visualizes only a deep arch that is not complete. Ulnar artery shows a corkscrew appearance. **Figure S7.** Patient with 12 years of vibration exposure and a prolonged Allen’s test ulnar side. The US indicated suspected radial stenosis, and the MRA visualizes only complete superficial arch and stenosis or incomplete ulnar part of the deep arch. **Figure S8.** Patient with 14 years of vibration exposure and Allen’s test is prolonged radially. The US showed spastic vessels and atypical anatomy. The MRA visualizes one arch (superficial) with atypical anatomy where three arteries (persistent median artery) supply the hand. **Figure S9.** A young patient with short exposure, Allen’s test prolonged radially, and the US showed only ulnar flow. The MRA visualizes a thin radial artery, with suspected vasospasm rather than stenosis in a segment of the only present arch (deep). **Figure S10.** A Patient with 20 years of vibration exposure and Allen’s test prolonged radially. The US showed only ulnar flow, and MRA (Rotated MIP picture) shows only deep arch together with incomplete superficial arch, and suspected stenosis that probably is a spastic segment in the arch.

## Data Availability

The data used in this study was derived from Patients’ data. Any researcher granted ethical approval from a regional ethical board can contact the Department of Environmental and Occupational Medicine at Örebro University Hospital (USÖ) for the study data. However, the Swedish National Board of Health and Welfare also places restrictions on sharing sensitive information.
