# Correction to: Arterial abnormalities in the hands of workers with vibration white fingers – a magnetic resonance angiography case series

**DOI:** 10.1186/s12995-021-00323-1

**Published:** 2021-08-11

**Authors:** Per Vihlborg, Karim Makdoumi, Hana Gavlovská, Sverre Wikström, Pål Graff

**Affiliations:** 1 Department of Occupational and Environmental Medicine, Faculty of Medicine and Health, Örebro University, SE 701 82 Örebro, Sweden; 2 Odensbackens Health Center, Örebro, Sweden; 3 Departement of geriatrics, Faculty of Medicine and Health, Örebro University, SE 701 82 Örebro, Sweden; 4 School of Medical Sciences, Örebro University, Örebro, Sweden; 5 Department of Ophthalmology, Faculty of Medicine and Health, Örebro University, Örebro, Sweden; 6 Department of radiology, Örebro University, Hospital, PO Box 1613, SE-701 16 Region Örebro County, Sweden; 7 National Institute of Occupational Health (STAMI), Oslo, Norway

**Correction to: J Occup Med Toxicol 16, 27 (2021)**

**https://doi.org/10.1186/s12995-021-00319-x**

Following the publication of the original article [1], we were notified that one author name has been incorrectly spelled.

Original spelling: Karim Makdomi

Correct spelling: Karim Makdoumi

The original article has now been corrected.
