# Supplementary material for: Arterial abnormalities in the hands of workers with vibration white fingers – a magnetic resonance angiography case series
Source: J Occup Med Toxicol. 2021 Jul 29;16:27. doi: 10.1186/s12995-021-00319-x (PMC8320041; doi:10.1186/s12995-021-00319-x)
Supplement: Supplementary file 1 — Additional file 1: Figure S1. Patient with prolonged Allen’s test on the ulnar side and 20 years of high vibration exposure. The ultrasound (US) showed suspected distal radial occlusion, and MRA shows only deep arch and stenosis in the junction with the ulnar artery. Ulnar artery shows a corkscrew appearance. Figure S2. Patient with 40 years of vibration exposure and prolonged Allen’s test on the radial. The US showed reduced flow from the radial artery MRA image quality was inadequate because of the size of the hands, making the use a wrist coil impossible instead examined in head coil. Ulnar artery shows a corkscrew appearance. Figure S3. Patient with prolonged Allen’s test on the ulnar side and 43 years of vibration exposure. US and MRA show an occlusion in the ulnar artery, and the MRA also shows a stenosis in the radial artery. Deep arch is present and a variant of superficial arch that originates from the deep arch. Figure S4. Patient with 30 years of vibration exposure and prolonged Allen’s test on the ulnar side. The US showed a thin ulnar artery. The MRA picture shows deep arch and stenosis in the junction of the deep arch to ulnar artery. Ulnar artery shows a corkscrew appearance. Figure S5. Patient with 22 years of low vibration exposure with Allen’s test prolonged ulnar. In this patient, the Allen’s test was difficult to perform. The compression of the wrist did not completely produce a paused blood supply. The US showed only ulnar flow, and MRA shows a variant with duplicated superficial arch and a normal deep arch. Figure S6. Patient with 14 years of vibration exposure and prolonged Allen’s test ulnar. The US indicated suspected a radial stenosis and the MRA visualizes only a deep arch that is not complete. Ulnar artery shows a corkscrew appearance. Figure S7. Patient with 12 years of vibration exposure and a prolonged Allen’s test ulnar side. The US indicated suspected radial stenosis, and the MRA visualizes only complete superficial arch and stenosis [file 12995_2021_319_MOESM1_ESM.docx]

Supplementfile

Magnetic Resoncance Angiography (MRA) pictures from the ten participants.


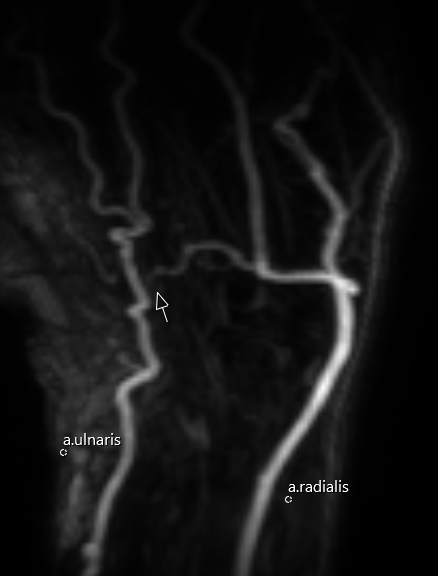

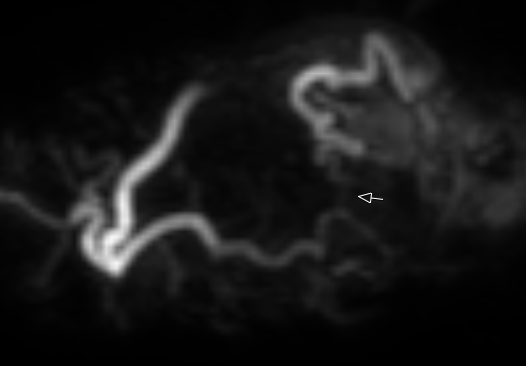


Figure 1. Patient with prolonged Allen's test on the ulnar side and 20 years of high vibration exposure. The ultrasound (US) showed suspected distal radial occlusion, and MRA shows only deep arch and stenosis in the junction with the ulnar artery. Ulnar artery shows a corkscrew appearance.


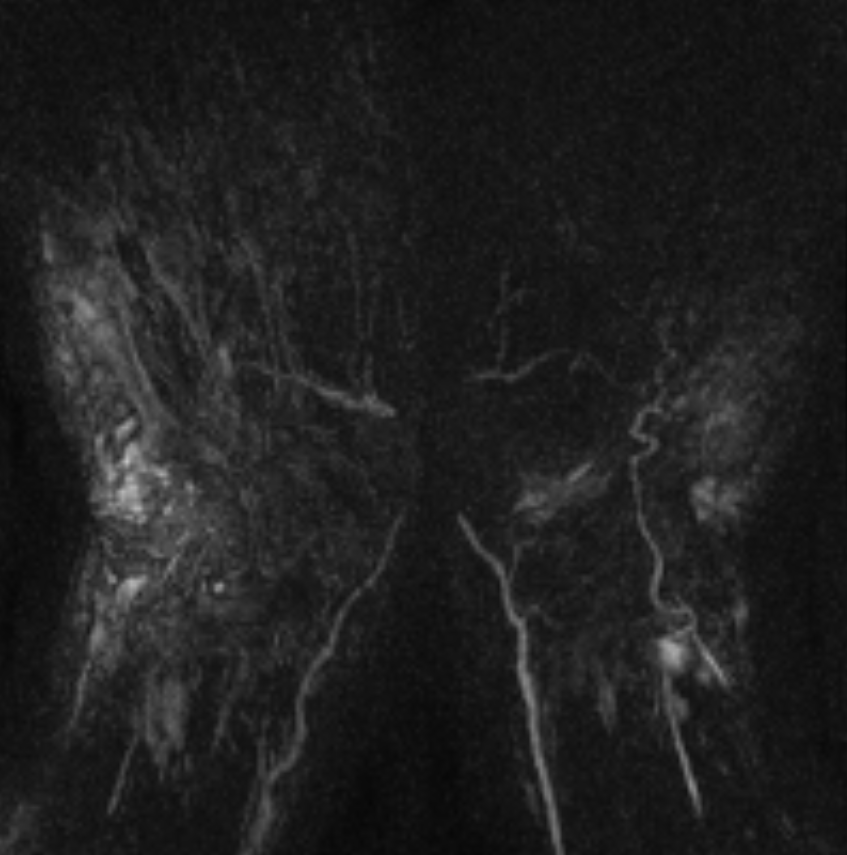


Figure 2. Patient with 40 years of vibration exposure and prolonged Allen's test on the radial. The US showed reduced flow from the radial artery MRA image quality was inadequate because of the size of the hands, making the use a wrist coil impossible instead examined in head coil. Ulnar artery shows a corkscrew appearance.


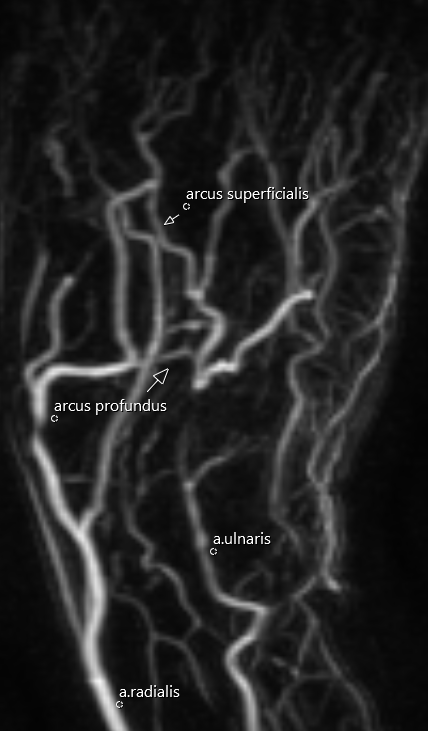

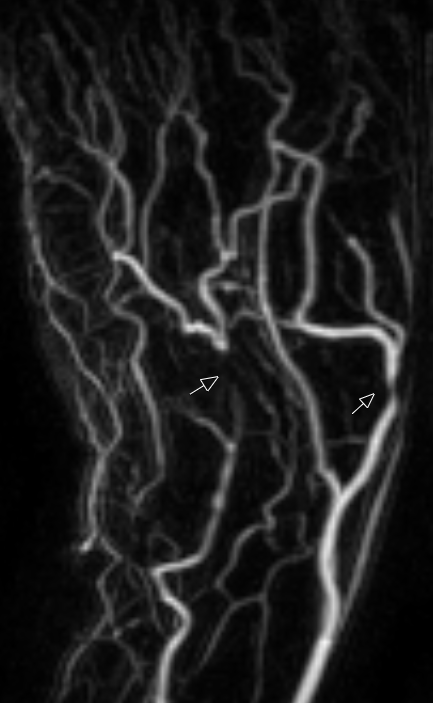


Figure 3. Patient with prolonged Allen's test on the ulnar side and 43 years of vibration exposure. US and MRA show an occlusion in the ulnar artery, and the MRA also shows a stenosis in the radial artery. Deep arch is present and a variant of superficial arch that originates from the deep arch.


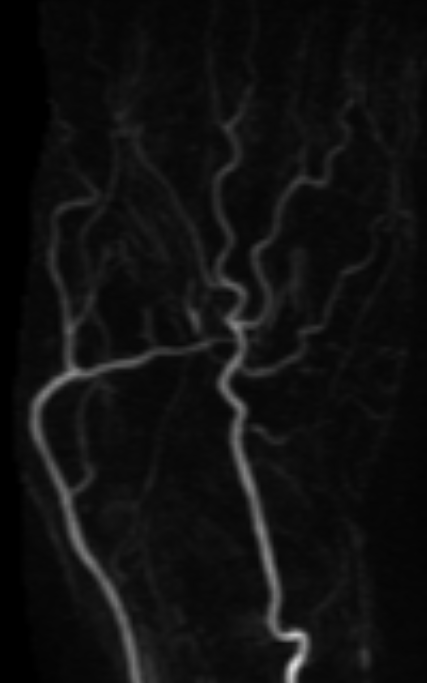


Figure 4. Patient with 30 years of vibration exposure and prolonged Allen's test on the ulnar side. The US showed a thin ulnar artery. The MRA picture shows deep arch and stenosis in the junction of the deep arch to ulnar artery. Ulnar artery shows a corkscrew appearance.


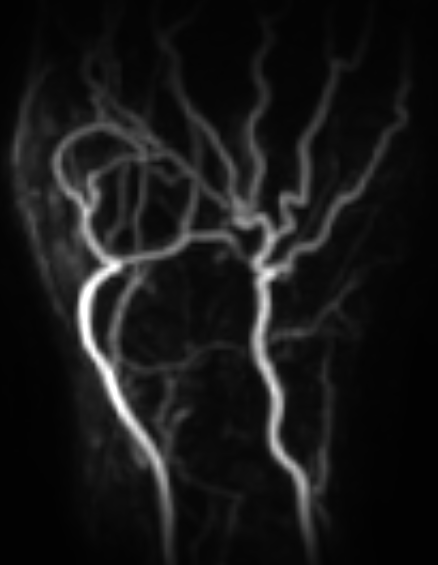


Figure 5. Patient with 22 years of low vibration exposure with Allen's test prolonged ulnar. In this patient, the Allen's test was difficult to perform. The compression of the wrist did not completely produce a paused blood supply. The US showed only ulnar flow, and MRA shows a variant with duplicated superficial arch and a normal deep arch.


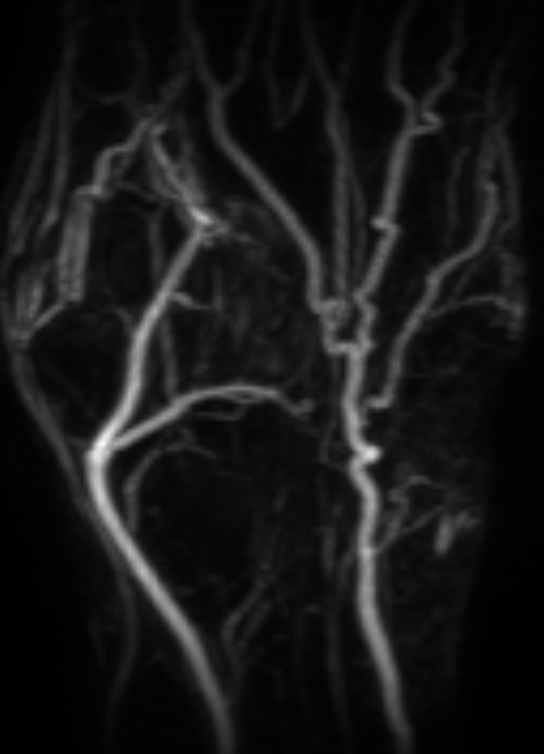

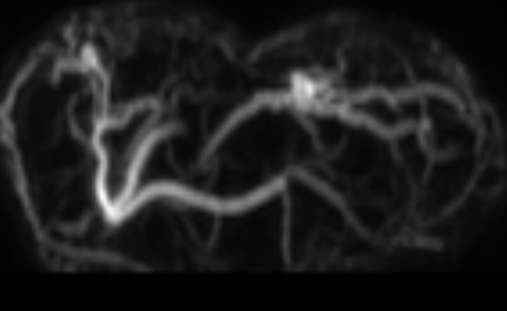


Figure 6. Patient with 14 years of vibration exposure and prolonged Allen’s test ulnar. The US indicated suspected a radial stenosis and the MRA visualizes only a deep arch that is not complete. Ulnar artery shows a corkscrew appearance.


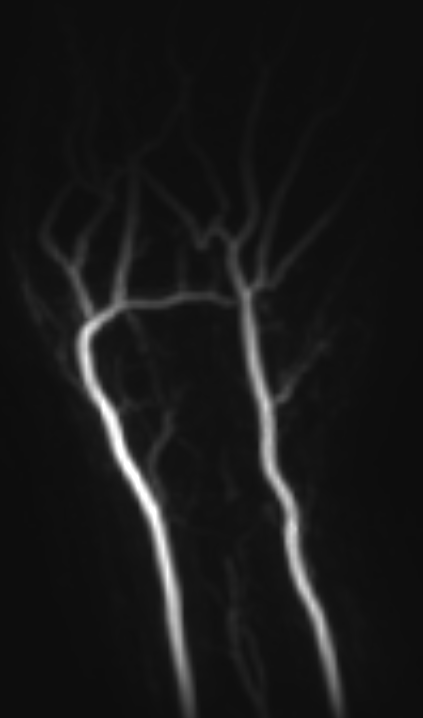


Figure 7. Patient with 12 years of vibration exposure and a prolonged Allen’s test ulnar side. The US indicated suspected radial stenosis, and the MRA visualizes only complete superficial arch and stenosis or incomplete ulnar part of the deep arch.


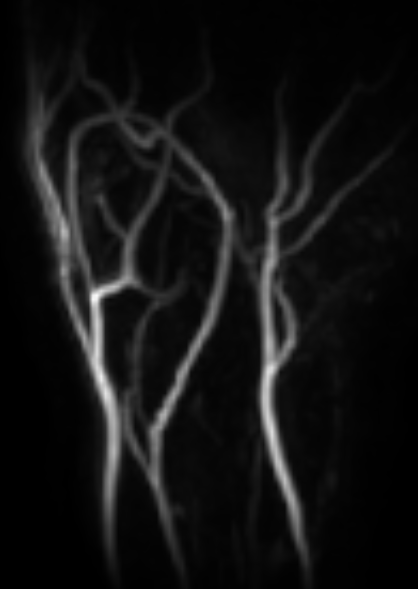


Figure 8. Patient with 14 years of vibration exposure and Allen’s test is prolonged radially. The US showed spastic vessels and atypical anatomy. The MRA visualizes one arch (superficial) with atypical anatomy where three arteries (persistent median artery) supply the hand.


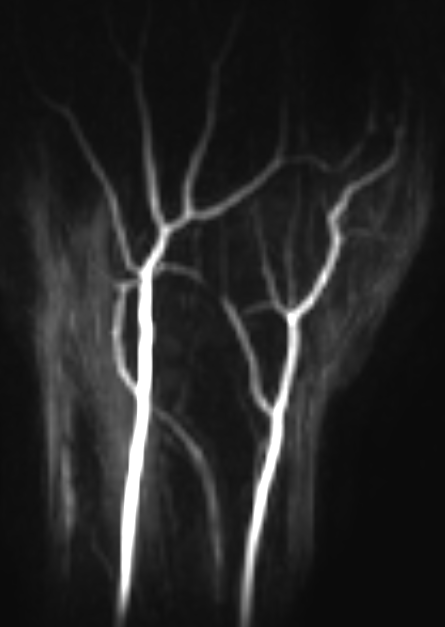

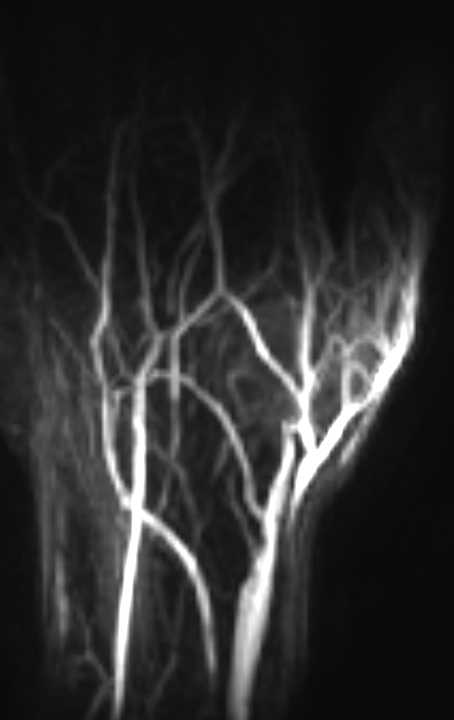


Figure 9. A young patient with short exposure, Allen's test prolonged radially, and the US showed only ulnar flow. The MRA visualizes a thin radial artery, with suspected vasospasm rather than stenosis in a segment of the only present arch (deep).


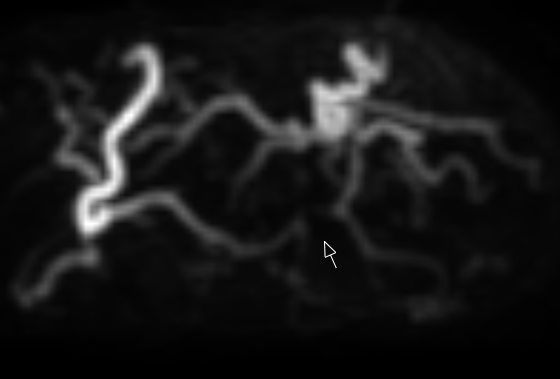

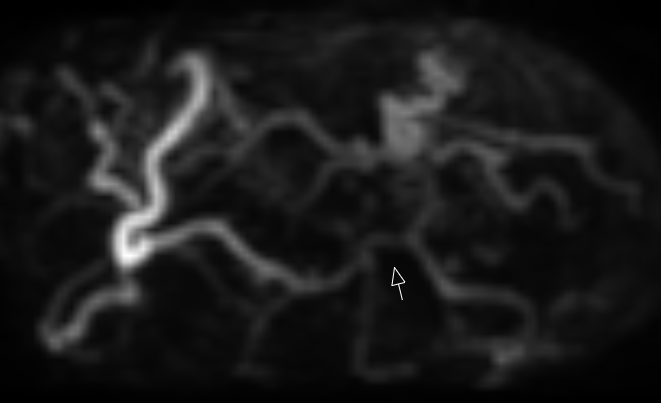


Figure 10

A Patient with 20 years of vibration exposure and Allen's test prolonged radially. The US showed only ulnar flow, and MRA (Rotated MIP picture) shows only deep arch together with incomplete superficial arch, and suspected stenosis that probably is a spastic segment in the arch.
